# Risk factor analysis and predictive model for the alleviation of postpartum stress urinary incontinence: a retrospective cohort study

**DOI:** 10.3389/fgwh.2026.1781403

**Published:** 2026-06-04

**Authors:** Lingdan Guo, Jueying Li, Jia Lu, Sijiao Chen, Mengying Li, Jingzhou Yang, Li Xiao, Jian Shen, Jianxin Liu

**Affiliations:** 1 Department of Ultrasound, The Central Hospital of Wuhan, Tongji Medical College, Huazhong University of Science and Technology, Wuhan, China; 2 Department of Obstetrics and Gynecology, The Central Hospital of Wuhan, Tongji Medical College, Huazhong University of Science and Technology, Wuhan, China

**Keywords:** body mass index, nomogram, pelvic floor muscle training, postpartum stress urinary incontinence, quality of life, risk factors

## Abstract

**Background:**

Postpartum stress urinary incontinence (SUI) can substantially affect women's daily functioning, psychosocial well-being, and rehabilitation needs. Predictive models for persistent postpartum SUI after basic pelvic floor muscle training (PFMT) remain limited. This study aimed to identify factors associated with persistent SUI and to develop a predictive nomogram for early risk stratification.

**Methods:**

This retrospective cohort study included 1,538 women diagnosed with SUI at one month postpartum who underwent basic PFMT and completed six-month follow-up at Wuhan Central Hospital between January 2020 and December 2024. Persistent SUI at six months postpartum was defined according to International Continence Society criteria as self-reported involuntary urine leakage during effort, exertion, sneezing, or coughing, confirmed during outpatient follow-up by clinical symptom assessment. Women without leakage symptoms at six months were classified into the Recovery Group, whereas women with ongoing leakage symptoms were classified into the Persistent SUI Group. Multivariable logistic regression was used to identify factors associated with persistent SUI. Model discrimination was evaluated using receiver operating characteristic (ROC) curve analysis. The optimal cutoff value was determined using the Youden index, and sensitivity, specificity, and accuracy were calculated. Internal validation was performed using 1,000 bootstrap resamples to estimate the apparent and optimism-corrected C-indexes. Calibration was assessed using a calibration curve and calibration slope, and clinical utility was evaluated using decision curve analysis (DCA). Nonlinear associations between postpartum BMI loss and persistent SUI were explored using restricted cubic splines and threshold analysis.

**Results:**

Among 1,538 women, 826 achieved symptom resolution and 722 had persistent SUI at six months postpartum. Multivariable analysis showed that older age, higher BMI, gestational diabetes mellitus, parity >=2, perineal tear, cystocele, uterine prolapse, rectocele, and levator ani hiatus defect were associated with higher odds of persistent SUI. Cesarean delivery and greater postpartum BMI loss were associated with lower odds of persistent SUI. The nomogram demonstrated good discrimination, with an AUC of 0.813 (95% CI: 0.792–0.834). At the optimal cutoff value of 0.549, sensitivity, specificity, and accuracy were 0.644, 0.828, and 0.742, respectively. Bootstrap validation yielded an apparent C-index of 0.813 and an optimism-corrected C-index of 0.808. The calibration slope was 0.974, and DCA suggested potential clinical net benefit across a range of threshold probabilities. A nonlinear U-shaped association was observed for postpartum BMI loss, with lower odds of persistent SUI below approximately 5 kg/m^2^ and higher odds above this threshold.

**Conclusion:**

Older age, higher BMI, gestational diabetes mellitus, higher parity, perineal trauma, pelvic organ prolapse, and levator ani hiatus defect were associated with persistent postpartum SUI after basic PFMT. Cesarean delivery and moderate postpartum BMI loss were associated with lower odds of persistent SUI. The nomogram showed favorable internal performance but requires external validation before routine clinical application. The nonlinear association between BMI loss and persistent SUI should be interpreted cautiously because residual confounding and unmeasured postpartum factors may have influenced this finding.

## Introduction

Stress urinary incontinence (SUI) is defined as involuntary urine leakage during activities that increase intra-abdominal pressure, including coughing, sneezing, laughing, or physical exertion (1). As one of the most prevalent forms of urinary incontinence, SUI affects a substantial proportion of women worldwide, with reported prevalence varying widely according to population characteristics, postpartum stage, and diagnostic criteria (2, 3). Postpartum women represent a particularly vulnerable population, especially during the early recovery period from one to six months after childbirth (4). A cohort study in China reported that approximately 25.9% of women experienced persistent SUI at 12 months postpartum (4). These findings indicate that postpartum SUI remains a clinically relevant women's health issue (5). Although postpartum SUI is not life-threatening, it can impair daily functioning, limit social participation, and negatively affect emotional well-being, sexual health, and overall quality of life. From a rehabilitation perspective, symptoms that are often considered mild may still lead to persistent functional impairment when early recognition and management are insufficient. Delayed intervention may increase long-term treatment burden and reduce the effectiveness of conservative therapies. Therefore, early identification of women at high risk of persistent SUI is of substantial clinical importance for optimizing postpartum rehabilitation strategies (6).

Multiple risk factors for SUI have been identified, including advanced maternal age, vaginal delivery, obesity, gestational diabetes mellitus, macrosomia, constipation, higher parity, and increased birth weight (7–9). These factors may contribute to structural and functional impairment of the pelvic floor through mechanical stretch, neuromuscular injury, and connective tissue damage. Older age, higher body mass index (BMI), and multiple vaginal deliveries have been consistently associated with increased risk and severity of SUI (10, 11). Beyond its physiological burden, SUI may also lead to embarrassment, social withdrawal, psychological distress, and increased socioeconomic burden (12, 13). Despite the recognition of these factors, their combined impact on symptom persistence following postpartum rehabilitation remains insufficiently characterized.

Pelvic floor muscle training (PFMT), particularly Kegel exercises, is a cornerstone of SUI treatment and prevention (14, 15). Previous studies have demonstrated that correct and sustained PFMT can result in clinically meaningful symptom improvement in a substantial proportion of women (16). Adjunctive therapies, such as biofeedback and electrical stimulation, may further enhance treatment response, especially among women with impaired pelvic floor muscle function (17). However, most existing studies have focused on overall symptom improvement rather than the identification of women at risk of persistent SUI after basic PFMT. This limitation is clinically relevant in routine clinical settings where advanced rehabilitation modalities are not universally accessible and where PFMT remains the primary intervention. At present, clinically applicable prediction tools for persistent postpartum SUI remain limited, particularly those designed to support early risk stratification in women undergoing basic PFMT. Early identification of high-risk individuals could facilitate personalized management strategies, including intensified pelvic floor rehabilitation, closer follow-up, and timely referral for specialized care. Furthermore, understanding modifiable factors, such as postpartum BMI changes, may provide additional opportunities for targeted intervention.

Therefore, this study aimed to evaluate outcomes after basic PFMT among women diagnosed with SUI at one month postpartum, to identify factors associated with persistent SUI at six months postpartum, and to develop and internally validate a predictive nomogram for early risk stratification and clinical decision support.

## Methods

### Study population

**Figure 1:**
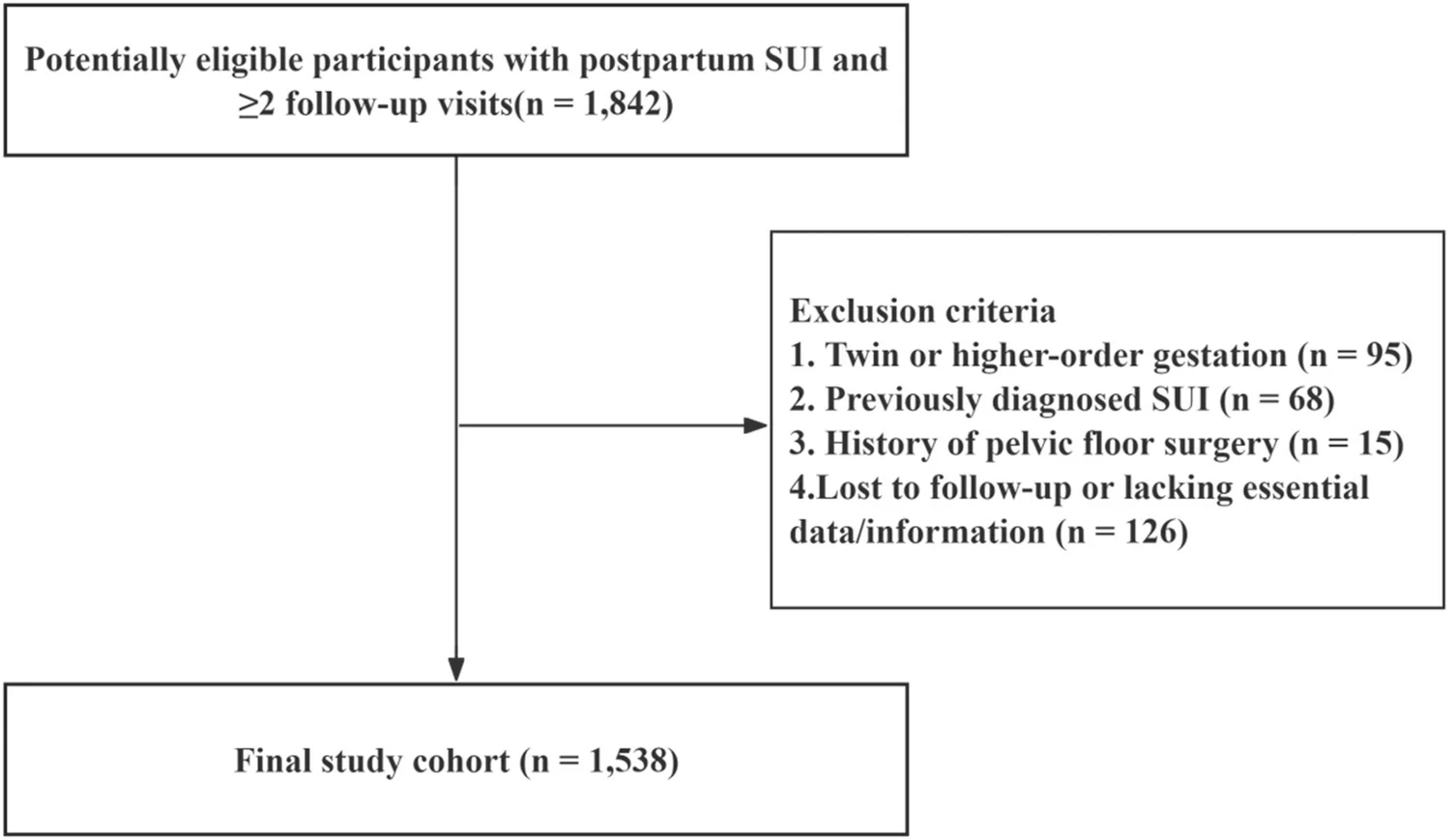
Flowchart of study population selection. SUI, Stress urinary incontinence.

This retrospective cohort study included women who delivered at Wuhan Central Hospital between January 2020 and December 2024 and completed postpartum follow-up visits at 1 month and 6 months postpartum. Eligible participants were aged 18 years or older, had complete medical records, were diagnosed with SUI at the 1-month postpartum visit, received basic PFMT as part of routine postpartum management, and attended both follow-up visits. Exclusion criteria were incomplete labor or follow-up data, twin or higher-order pregnancies (*n* = 95), pre-existing SUI before pregnancy (*n* = 68), previous pelvic floor surgery (*n* = 15), and loss to follow-up or missing critical data (*n* = 126). In total, 1,538 women were included in the final analysis (Figure 1). The study was approved by the Ethics Committee of Wuhan Central Hospital (Approval No. WHZXKYL2024-251) and was conducted in accordance with the Declaration of Helsinki. Because this was a retrospective study using de-identified clinical data and involving no additional intervention, the requirement for written informed consent was waived (18).

### Data collection and definitions

Data were extracted from the hospital's electronic medical records. Demographic variables included age, medical history, and gestational age at delivery. Pre-pregnancy weight was obtained from medical records or routine obstetric registration records, and height was measured with participants wearing light clothing and no shoes. BMI was calculated as weight in kilograms divided by height in meters squared (kg/m^2^). Postpartum BMI loss was calculated as pre-pregnancy BMI minus BMI at 1 month postpartum, expressed in kg/m^2^. Therefore, a higher value indicated a greater reduction in BMI by 1 month postpartum. Waist circumference was measured at the midpoint between the lower rib margin and the iliac crest, and abdominal circumference was measured at the level of the umbilicus; both were recorded to the nearest 0.1 cm. Uterine height was measured from the pubic symphysis to the uterine fundus. Neonatal weight and head circumference were measured using standard scales and measuring tapes. Blood pressure was measured using an automated sphygmomanometer (Omron HBP-9020). The average of two readings from the arm with the higher initial value was recorded, and a third reading was obtained if the difference exceeded 5 mmHg. Fasting blood glucose and glycated hemoglobin were measured after an 8-hour fast.

Hypertensive disorders of pregnancy were defined according to the American College of Obstetricians and Gynecologists (2020) criteria as blood pressure ≥140/90 mmHg on two occasions at least 4 h apart after 20 weeks of gestation (19). Gestational diabetes mellitus was diagnosed according to the American Diabetes Association (2021) criteria, defined as fasting blood glucose ≥92 mg/dL or 2-hour blood glucose ≥153 mg/dL during a 75-g oral glucose tolerance test (20, 21).

To ensure adequate statistical power and model stability, the sample size was evaluated based on the rule of 10 events per variable (EPV) for logistic regression, as recommended for robust multivariable modeling (22, 23). With 11 covariates planned for inclusion in the primary analysis, at least 110 outcome events were required. The final sample included 720 persistent SUI events, exceeding this threshold and supporting sufficient statistical power and acceptable model stability.

### SUI diagnosis

SUI was defined according to the International Continence Society as involuntary urine leakage during effort, exertion, sneezing, or coughing (24). At the 1-month postpartum visit, SUI diagnosis was based on patient-reported leakage symptoms consistent with this definition and clinical confirmation during postpartum outpatient assessment. The primary outcome was persistent SUI at 6 months postpartum. Women who continued to report involuntary leakage during effort, exertion, sneezing, or coughing at the 6-month follow-up were classified into the Persistent SUI Group, whereas women who reported complete resolution of leakage symptoms were classified into the Recovery Group. Because standardized pad tests or validated SUI severity questionnaires were not routinely available for all participants in this retrospective dataset, the outcome reflected clinically assessed symptom persistence rather than objectively quantified leakage severity.

### Pelvic floor muscle training protocol

All women diagnosed with SUI at the one-month postpartum visit received basic PFMT as part of routine postpartum management. Before the initiation of training, a clinician provided face-to-face instruction on pelvic floor anatomy, identification of the pelvic floor muscles, and the correct technique for voluntary pelvic floor contraction. Participants were specifically instructed to avoid inappropriate compensatory maneuvers, such as excessive abdominal straining, gluteal contraction, or breath-holding during pelvic floor contraction. Following the initial teaching session, women were encouraged to continue PFMT at home. To enhance supervision and engagement, weekly WeChat check-ins were used to remind participants to continue training and to monitor their participation. In addition, all participants were scheduled for monthly outpatient follow-up visits, during which symptom changes were reviewed, pelvic floor contraction performance was reassessed, and further education or correction was provided when necessary. This basic PFMT management pathway was maintained throughout follow-up until 6 months postpartum.

Because this was a retrospective study based on routine clinical practice, detailed quantitative data on PFMT frequency, session duration, and exact completion rates were not consistently available for all participants. Therefore, PFMT adherence was managed through WeChat check-ins and outpatient follow-up records, but could not be evaluated using a fully standardized quantitative adherence metric. Variation in actual PFMT dose, adherence, and performance quality may have contributed to differences in symptom persistence and was therefore considered an important methodological limitation.

### Pelvic floor ultrasound measurements

#### Transperineal ultrasound (TPUS)

Pelvic floor parameters were assessed using three-dimensional transperineal ultrasound (TPUS) with a GE Voluson E8 system (RIC 5–9-D probe, 5–10 MHz). Participants were examined in the supine lithotomy position after voiding, with a residual urine volume of less than 50 mL. A maximal and sustained Valsalva maneuver was performed by asking the participant to bear down as if having a bowel movement, and adequacy was visually confirmed by significant descent of the pelvic organs (25, 26).

#### Measurement methods

- **Post-void residual (PVR) urine volume (mL):** measured by transabdominal ultrasound immediately after spontaneous voiding.
- **Detrusor muscle thickness (cm):** measured from the anterior bladder wall using transabdominal ultrasound with the bladder filled to approximately 200–300 mL.
- **Posterior urethrovesical angle (degrees):** measured as the angle between the posterior bladder wall and the proximal urethra on a midsagittal transperineal ultrasound image at rest.
- **Organ positions (bladder neck, cervix, and rectal ampulla) (cm):** measured as the vertical distance from the inferior border of the pubic symphysis to the most caudal portion of each structure on a midsagittal transperineal ultrasound image at rest.
- **Valsalva measurements (posterior urethrovesical angle, urethral rotation angle, bladder neck position, bladder lowest point, cervix position, and rectal ampulla position):** measured using the same method as at rest, but during sustained maximal Valsalva.
- **Urethral rotation angle (degrees):** measured as the angle between the vertical line and the axis of the proximal urethra during maximal Valsalva.
- **Bladder lowest point (cm):** measured as the vertical distance from the inferior border of the pubic symphysis to the lowest point of the bladder during maximal Valsalva.
- **Valsalva levator hiatus area (cm^2^):** measured by tracing the hyperechoic rim of the levator ani muscle in the axial plane during maximal Valsalva, typically using 3D/4D ultrasound volume rendering.
- **Bladder neck descent (cm):** calculated as the difference in bladder neck position between rest and maximal Valsalva.
- **Bladder neck mobility [*n* (%)]:** defined as present when bladder neck descent was ≥1.5 cm during Valsalva or when there was clear visual evidence of marked bladder neck descent beyond the pubic symphysis.
- **Cystocele, uterine prolapse, and rectocele [*n* (%)]:** assessed clinically using the Pelvic Organ Prolapse Quantification (POP-Q) system. Prolapse was considered present when the leading edge of the bladder (point Ba), cervix/cuff (point C), or rectum (point Rp) was at or beyond the hymen (POP-Q stage ≥2).
- **Levator ani hiatus defect [*n* (%)]:** defined as levator avulsion, diagnosed as discontinuity or detachment of the puborectalis muscle from its insertion on the pubic bone on 3D/4D transperineal ultrasound axial images.
- **Bladder neck funneling [*n* (%)]:** defined as present when the bladder neck opened into a funnel shape >0.5 cm in length during Valsalva on transperineal ultrasound.

### Statistical analysis

Continuous variables were presented as mean ± standard deviation (SD) and compared using Welch's *t*-test. Categorical variables were presented as frequencies and percentages and compared using Pearson's chi-squared test. Outliers were handled by winsorization at the 1st and 99th percentiles. Missing data (<20%) were imputed using the random forest method. Variables with *P* < 0.1 in univariate analysis were entered into multivariable logistic regression to identify independent predictors. Clinically important variables were also considered when constructing the final model to reduce the risk of excluding relevant covariates solely because of univariate screening.

A nomogram and receiver operating characteristic (ROC) curve were constructed to evaluate model performance. Model discrimination was assessed using the area under the ROC curve (AUC). The optimal cutoff value was determined using the Youden index, and sensitivity, specificity, and accuracy were calculated. Internal validation was performed using 1,000 bootstrap resamples. Model calibration was assessed using a calibration curve and calibration slope. Nonlinear associations between postpartum BMI loss and persistent SUI were examined using restricted cubic splines (RCS). Threshold effects were further evaluated using two-piecewise linear regression models and likelihood ratio tests. All statistical analyses were performed using R version 4.3.4, and a two-sided *P* < 0.05 was considered statistically significant.

## Results

### Baseline characteristics

Among the 1,538 women diagnosed with SUI at 1 month postpartum who underwent PFMT, 826 achieved symptom resolution by 6 months postpartum and were classified into the Recovery Group, whereas 722 had persistent SUI and were classified into the Persistent SUI Group. Compared with women in the Recovery Group, those in the Persistent SUI Group were older, had a higher maternal BMI, and showed higher gravidity and parity. Vaginal delivery was more common in the Persistent SUI Group, whereas cesarean delivery was more frequent in the Recovery Group. The Persistent SUI Group also had higher incidences of gestational diabetes mellitus and perineal tear. In contrast, women in the Recovery Group showed a greater reduction in BMI from pre-pregnancy to 1 month postpartum. Pelvic organ prolapse assessments showed that the Persistent SUI Group had significantly higher rates of cystocele, uterine prolapse, rectocele, and levator ani muscle defects/levator hiatus abnormalities. Detailed baseline characteristics are presented in Table 1.

**Table 1:** Baseline characteristics of study participants.

Variable	Recover group (*N* = 818)	SUI group (*N* = 720)	*P-value*
Maternal Characteristics			
Maternal Age, Mean (SD)	29.76 (6.32)	30.71 (6.31)	0.003
Gestational Age at Delivery, Mean (SD)	38.51 (0.98)	38.54 (1.05)	0.561
Maternal Body Mass Index (BMI), Mean (SD)	25.61 (2.82)	26.26 (2.75)	<0.001
Maternal Abdominal Circumference, Mean (SD)	99.90 (5.98)	100.30 (5.74)	0.171
Fundal Height, Mean (SD)	32.96 (1.96)	33.06 (1.95)	0.301
Mode of Delivery, *n* (%)			<0.001
Vaginal Delivery	456 (56%)	588 (82%)	
Cesarean Section	362 (44%)	132 (18%)	
Gravidity, *n* (%)			0.035
1	691 (84%)	572 (79%)	
2	94 (11%)	107 (15%)	
≥3	33 (4%)	41 (6%)	
Parity, *n* (%)			0.006
1	763 (93%)	642 (89%)	
≥2	55 (7%)	78 (11%)	
Gestational Hypertension, *n* (%)	34 (4%)	42 (6%)	0.162
Gestational Diabetes Mellitus, *n* (%)	54 (7%)	76 (11%)	0.007
Perineal Tear, *n* (%)	360 (44%)	438 (61%)	<0.001
Newborn Characteristics			
Newborn Birth Weight, Mean (SD)	3.25 (0.38)	3.27 (0.40)	0.361
Newborn Head Circumference, Mean (SD)	33.32 (0.76)	33.35 (0.82)	0.581
Newborn Abdominal Circumference, Mean (SD)	33.66 (1.44)	33.60 (1.54)	0.431
Postpartum Measurements			
Postpartum BMI Loss, Mean (SD)	4.58 (1.25)	3.86 (1.31)	<0.001
Post-void Residual Volume (PVR), Mean (SD), mL	12.31 (13.51)	11.54 (13.25)	0.261
Detrusor Muscle Thickness, Mean (SD), cm	0.31 (0.05)	0.31 (0.05)	0.201
Posterior Urethrovesical Angle (PUVA), Mean (SD), degrees	97.38 (19.57)	97.52 (18.32)	0.891
Bladder Neck Position, Mean (SD), cm	2.42 (0.33)	2.42 (0.33)	0.751
Cervix Position, Mean (SD), cm	3.13 (0.80)	3.14 (0.78)	0.871
Rectal Ampulla Position, Mean (SD), cm	1.31 (0.57)	1.30 (0.58)	0.851
Valsalva PUVA, Mean (SD), degrees	122.81 (33.89)	123.64 (33.14)	0.621
Valsalva Urethral Rotation Angle, Mean (SD), degrees	48.55 (36.55)	49.16 (39.00)	0.751
Valsalva Bladder Neck Position, Mean (SD), cm	0.73 (1.10)	0.75 (1.06)	0.661
Valsalva Bladder Lowest Point, Mean (SD), cm	0.72 (1.03)	0.71 (1.03)	0.811
Valsalva Cervix Position, Mean (SD), cm	1.46 (1.20)	1.42 (1.21)	0.481
Valsalva Rectal Ampulla Position, Mean (SD), cm	0.21 (0.76)	0.17 (0.77)	0.281
Valsalva Levator Hiatus Area, Mean (SD), cm²	20.56 (4.96)	20.52 (5.08)	0.861
Bladder Neck Descent, Mean (SD), cm	1.70 (1.07)	1.66 (1.07)	0.501
Bladder Neck Mobility, *n* (%)	309 (38%)	291 (40%)	0.312
Cystocele, *n* (%)	214 (26%)	378 (53%)	<0.001
Uterine Prolapse, *n* (%)	152 (19%)	302 (42%)	<0.001
Rectocele, *n* (%)	28 (3%)	78 (11%)	<0.001
Levator Ani Hiatus Defect, *n* (%)	336 (41%)	483 (67%)	<0.001
Bladder Neck Funneling, *n* (%)	108 (13%)	100 (14%)	0.752

Data are presented as mean (SD) for continuous variables and *n* (%) for categorical variables. *P*-values were calculated using Welch's *t*-test for continuous variables and Pearson's chi-squared test for categorical variables. Measurements denoted with “Valsalva” indicate values obtained during maximal Valsalva. Postpartum BMI loss was calculated as pre-pregnancy BMI minus BMI at 1 month postpartum. SUI, stress urinary incontinence; BMI, body mass index; PUVA, posterior urethrovesical angle.

### Univariate and multivariate logistic regression analysis

Multivariable logistic regression identified several independent factors associated with persistent SUI after PFMT (Table 2). In the final model, older age, higher BMI, gestational diabetes mellitus, parity ≥2, perineal tear, cystocele, uterine prolapse, rectocele, and levator ani hiatus defect were associated with higher odds of persistent SUI, whereas cesarean delivery and greater postpartum BMI loss were associated with lower odds of persistent SUI. Because this was a retrospective observational study, these associations should not be interpreted as evidence of causal protective or risk effects.

**Table 2:** Factors associated with persistent SUI after PFMT.

**Variable**	** Univariable analysis OR (95% CI, *P*-value)**	**Multivariable analysis OR (95% CI, *P*-value)**	**Final model OR (95% CI, *P*-value)**
Maternal Characteristics			
Maternal Age	1.02 (1.01–1.04, *P* = 0.003)	1.03 (1.01–1.05, *P* = 0.008)	1.03 (1.01–1.05, *P* = 0.008)
Gestational Age at Delivery	1.03 (0.93–1.14, *P* = 0.554)		
Maternal Body Mass Index (BMI)	1.09 (1.05–1.13, *P* < 0.001)	1.09 (1.04–1.14, *P* < 0.001)	1.09 (1.04–1.14, *P* < 0.001)
Maternal Abdominal Circumference	1.01 (0.99–1.03, *P* = 0.175)		
Fundal Height	1.03 (0.98–1.08, *P* = 0.302)		
Mode of Delivery (Ref: Vaginal Delivery)	0.28 (0.22–0.36, *P* < 0.001)	0.26 (0.20–0.34, *P* < 0.001)	0.26 (0.20–0.34, *P* < 0.001)
Gravidity (Ref: 1)			
2	1.38 (1.02–1.85, *P* = 0.036)	1.26 (0.81–1.96, *P* = 0.295)	
≥3	1.50 (0.94–2.41, *P* = 0.091)	1.06 (0.55–2.03, *P* = 0.857)	
Parity ≥2(Ref: 1)	1.69 (1.18–2.42, *P* = 0.005)	1.36 (0.77–2.41, *P* = 0.290)	1.60 (1.03–2.48, *P* = 0.035)
Gestational Hypertension (Ref: No)	1.43 (0.90–2.27, *P* = 0.132)		
Gestational Diabetes Mellitus (Ref: No)	1.67 (1.16–2.40, *P* = 0.006)	1.95 (1.27–3.00, *P* = 0.002)	1.93 (1.26–2.97, *P* = 0.002)
Perineal Tear (Ref: No)	1.98 (1.61–2.42, *P* < 0.001)	2.04 (1.60–2.59, *P* < 0.001)	2.04 (1.61–2.60, *P* < 0.001)
Newborn Characteristics			
Newborn Birth Weight	1.13 (0.87–1.46, *P* = 0.354)		
Newborn Head Circumference	1.04 (0.91–1.18, *P* = 0.575)		
Newborn Abdominal Circumference	0.97 (0.91–1.04, *P* = 0.428)		
Postpartum Measurements			
Postpartum BMI Loss	0.64 (0.59–0.70, *P* < 0.001)	0.64 (0.58–0.71, *P* < 0.001)	0.64 (0.59–0.71, *P* < 0.001)
Post-void Residual Volume (PVR)	1.00 (0.99–1.00, *P* = 0.262)		
Detrusor Muscle Thickness	0.27 (0.04–2.02, *P* = 0.202)		
Posterior Urethrovesical Angle (PUVA)	1.00 (1.00–1.01, *P* = 0.889)		
Bladder Neck Position	1.05 (0.78–1.43, *P* = 0.746)		
Cervix Position	1.01 (0.89–1.15, *P* = 0.872)		
Rectal Ampulla Position	0.98 (0.83–1.17, *P* = 0.854)		
Valsalva PUVA	1.00 (1.00–1.00, *P* = 0.624)		
Valsalva Urethral Rotation Angle	1.00 (1.00–1.00, *P* = 0.752)		
Valsalva Bladder Neck Position	1.02 (0.93–1.12, *P* = 0.660)		
Valsalva Bladder Lowest Point	0.99 (0.90–1.09, *P* = 0.807)		
Valsalva Cervix Position	0.97 (0.89–1.05, *P* = 0.476)		
Valsalva Rectal Ampulla Position	0.93 (0.82–1.06, *P* = 0.284)		
Valsalva Levator Hiatus Area	1.00 (0.98–1.02, *P* = 0.856)		
Bladder Neck Descent	0.97 (0.88–1.06, *P* = 0.499)		
Cystocele (Ref: No)	3.12 (2.52–3.86, *P* < 0.001)	2.14 (1.65–2.78, *P* < 0.001)	2.15 (1.65–2.78, *P* < 0.001)
Uterine Prolapse (Ref: No)	3.17 (2.51–3.98, *P* < 0.001)	2.10 (1.58–2.78, *P* < 0.001)	2.11 (1.59–2.79, *P* < 0.001)
Rectocele (Ref: No)	3.43 (2.20–5.34, *P* < 0.001)	3.39 (2.03–5.64, *P* < 0.001)	3.40 (2.04–5.65, *P* < 0.001)
Levator Ani Hiatus Defect (Ref: No)	2.92 (2.37–3.60, *P* < 0.001)	1.99 (1.54–2.57, *P* < 0.001)	1.98 (1.54–2.55, *P* < 0.001)
Bladder Neck Funneling (Ref: No)	1.06 (0.79–1.42, *P* = 0.695)		

OR, Odds Ratio; CI, Confidence Interval.

Reference categories are specified in parentheses for categorical variables.

### Prediction model and performance

**Figure 2:**
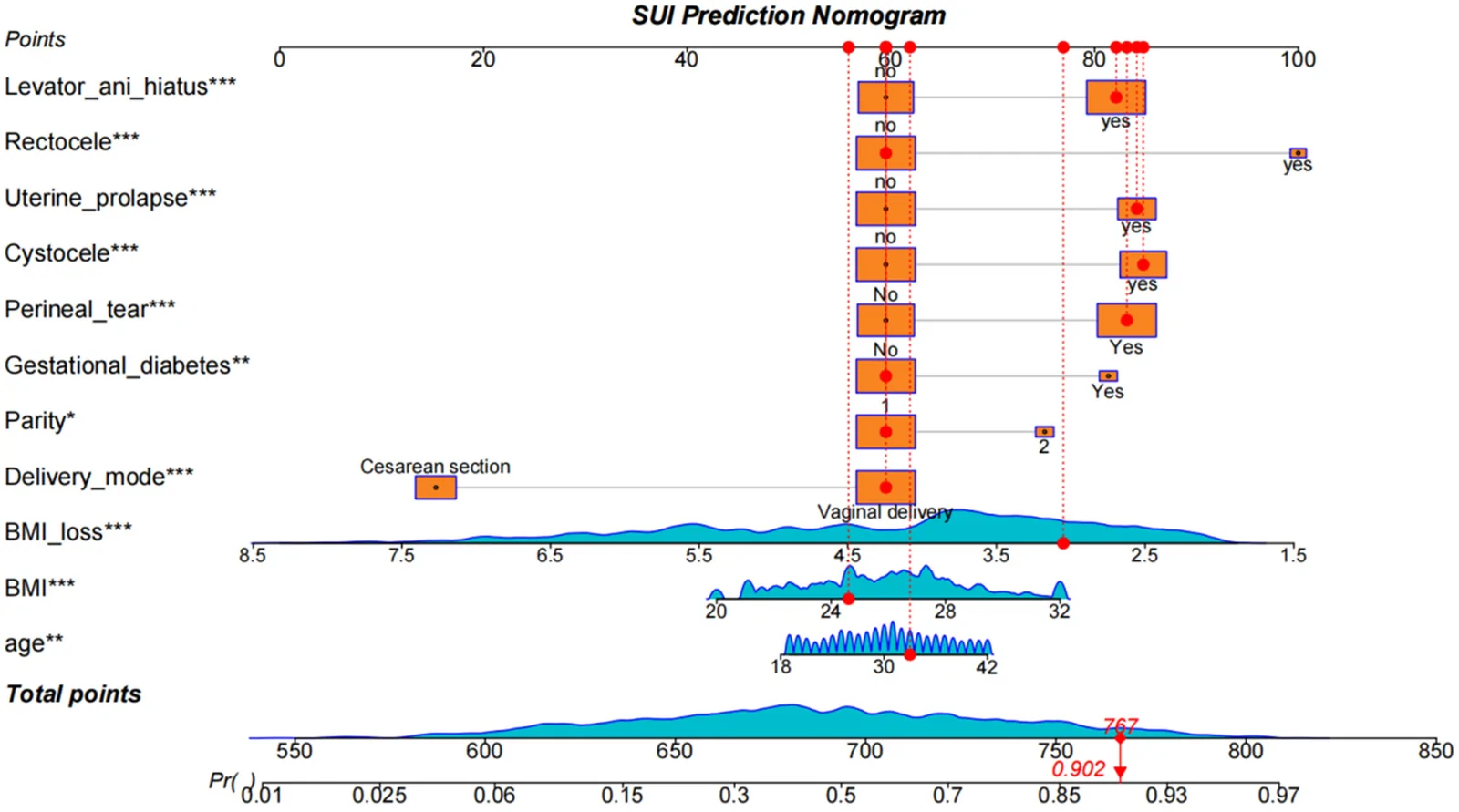
Nomogram for Prediction of Persistent Postpartum SUI. SUI, Stress Urinary Incontinence.

**Figure 3:**
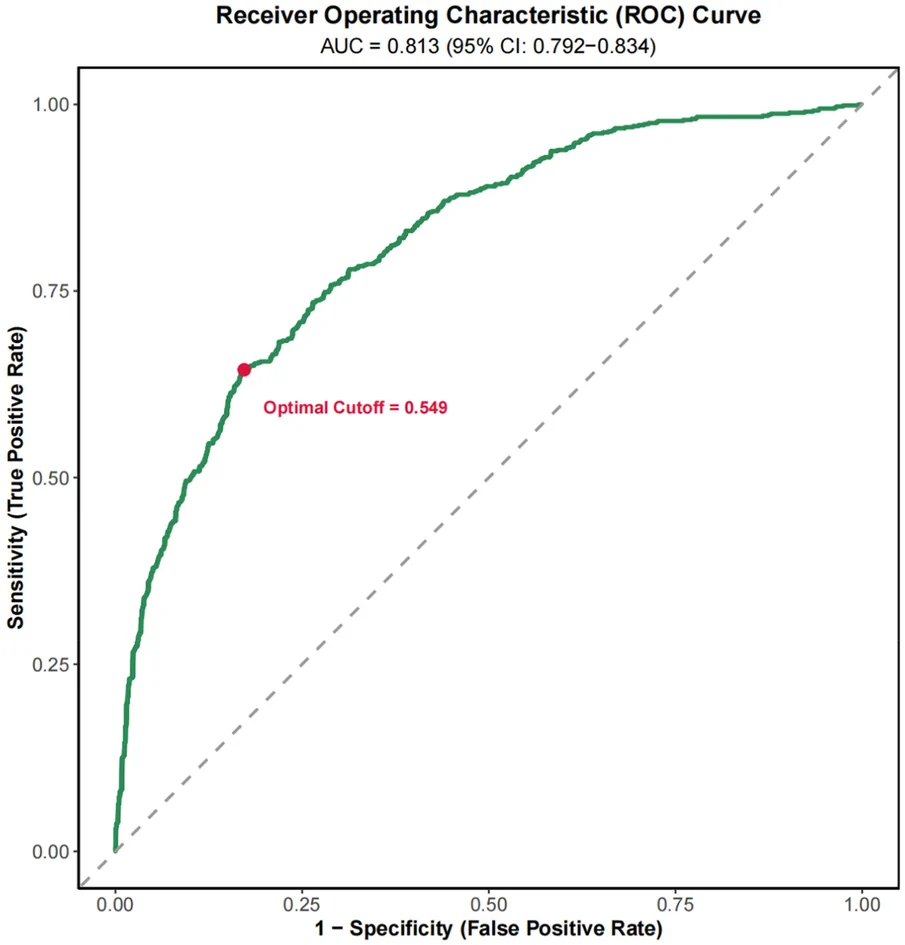
ROC Curve for Prediction of Persistent Postpartum SUI. SUI, Stress urinary incontinence; ROC, receiver operating characteristic.

**Figure 4:**
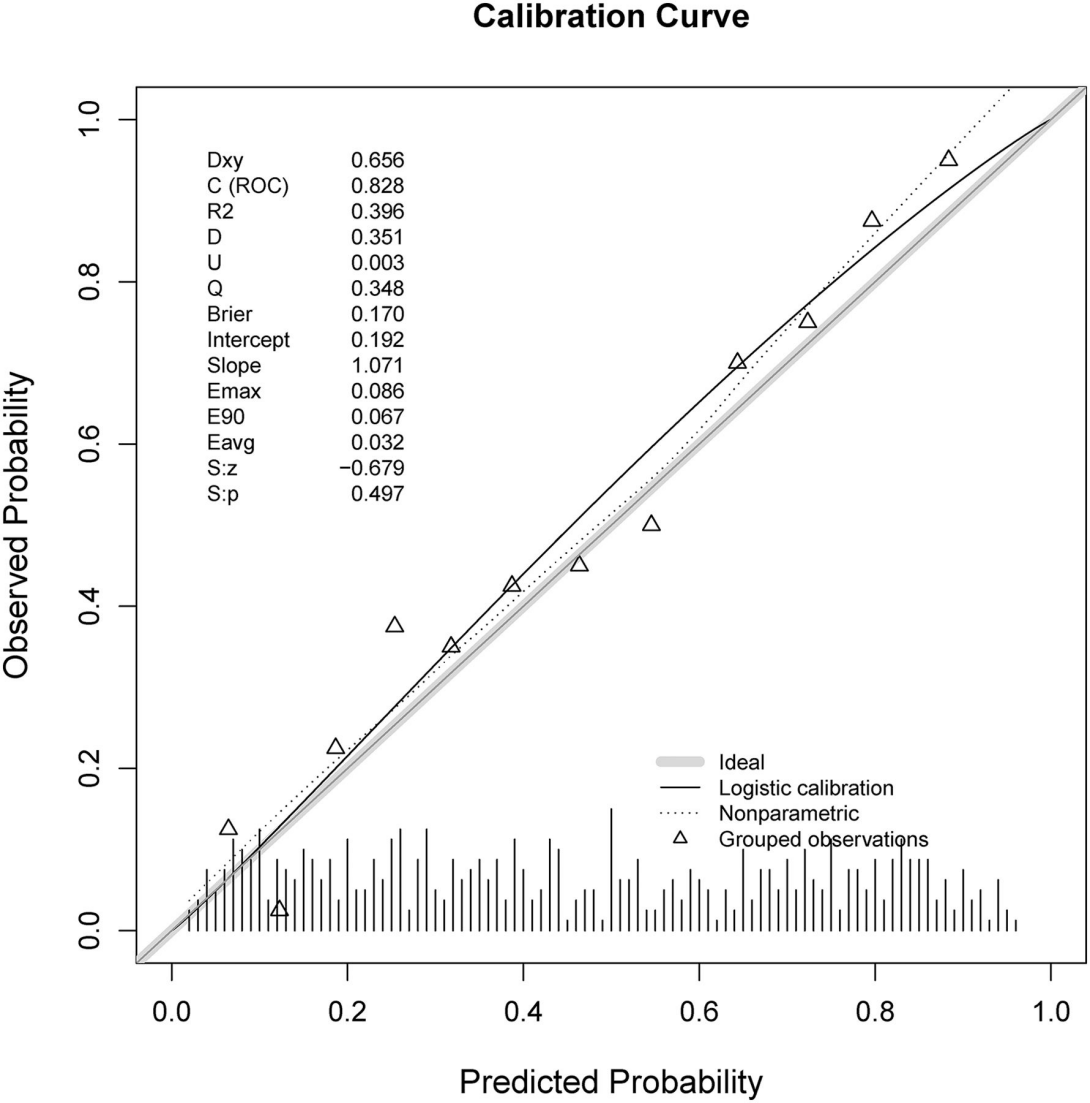
Calibration curve of the prediction model for persistent postpartum stress urinary incontinence.

**Figure 5:**
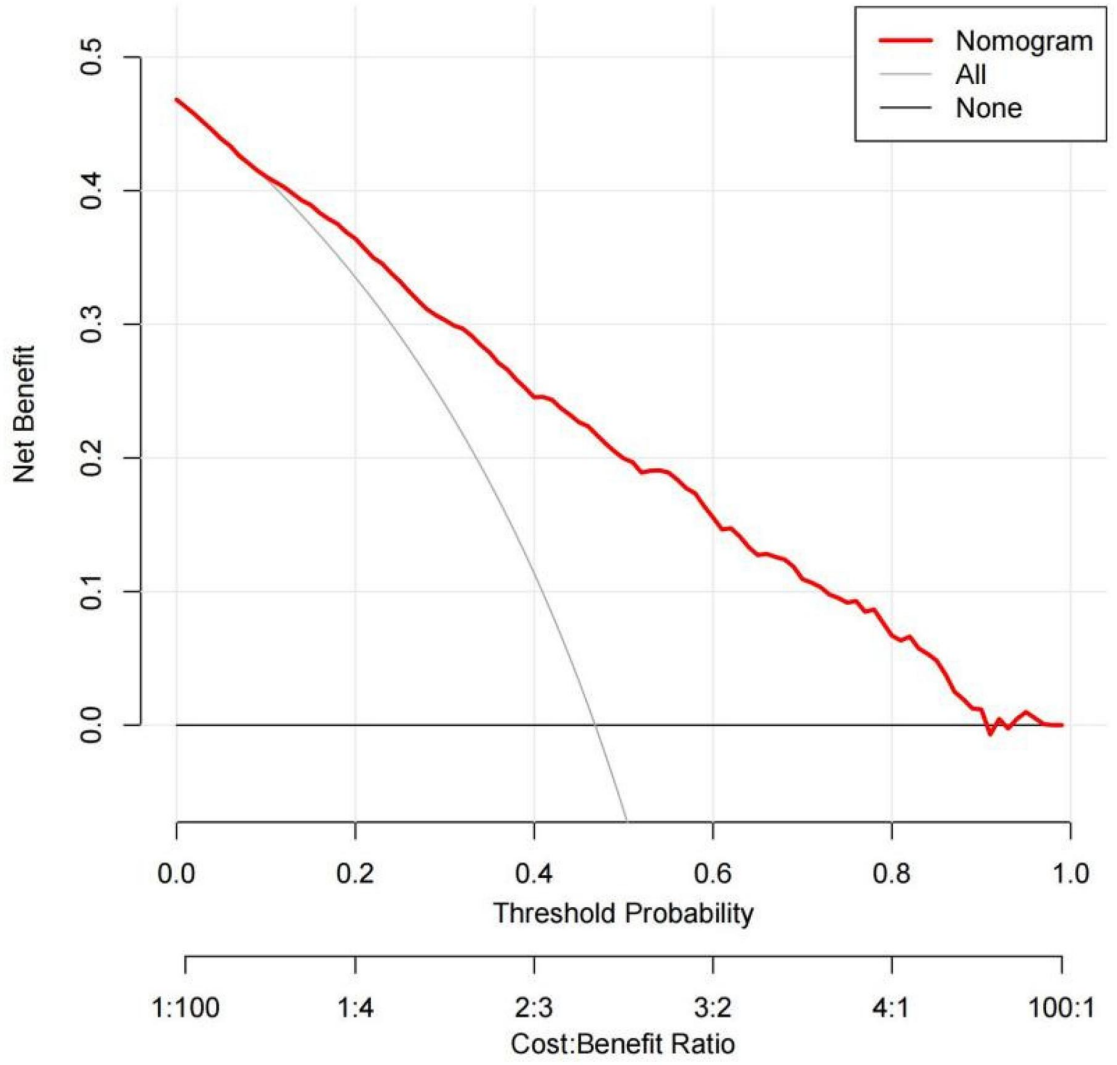
Decision curve analysis of the nomogram for predicting persistent postpartum stress urinary incontinence.

A nomogram was developed using significant variables from multivariate analysis, including age, BMI, gestational diabetes, perineal tear, posterior urethrovesical angle, cystocele, uterine prolapse, levator ani injury, cesarean section, and BMI loss (Figure 2). The model demonstrated good discriminative ability, with an area under the curve (AUC) of 0.813 (95% CI: 0.792–0.834). At the optimal cutoff value of 0.549, the model achieved a sensitivity of 0.644, a specificity of 0.828, and an accuracy of 0.742 (Figure 3). Internal validation using 1,000 bootstrap resamples showed stable model performance, with an apparent C-index of 0.813 and a bootstrap-corrected C-index of 0.808, indicating minimal optimism (0.005). The calibration slope was 0.974, suggesting minimal overfitting. The calibration curve (Figure 4) demonstrated good agreement between predicted and observed probabilities of persistent postpartum stress urinary incontinence, indicating satisfactory calibration performance. Decision curve analysis (Figure 5) showed that the nomogram provided a higher net benefit than both the treat-all and treat-none strategies across a wide range of threshold probabilities, indicating favorable clinical utility.

This figure presents the odds ratios (ORs) and their 95% confidence intervals for persistent SUI as a function of postpartum BMI loss. Postpartum BMI loss was calculated as pre-pregnancy BMI minus BMI at 1 month postpartum; therefore, higher values indicate greater BMI reduction by 1 month postpartum. A restricted cubic spline with 4 knots was used to capture the potential nonlinear relationship. The model was adjusted for key covariates, including maternal age, baseline BMI, mode of delivery, parity, gestational diabetes mellitus, perineal tear, cystocele, uterine prolapse, rectocele, and levator ani hiatus defect. The test for nonlinearity was significant (*P* < 0.001), indicating a nonlinear association between postpartum BMI loss and persistent SUI. SUI: stress urinary incontinence.

### Threshold effect analysis of BMI loss

**Figure 6:**
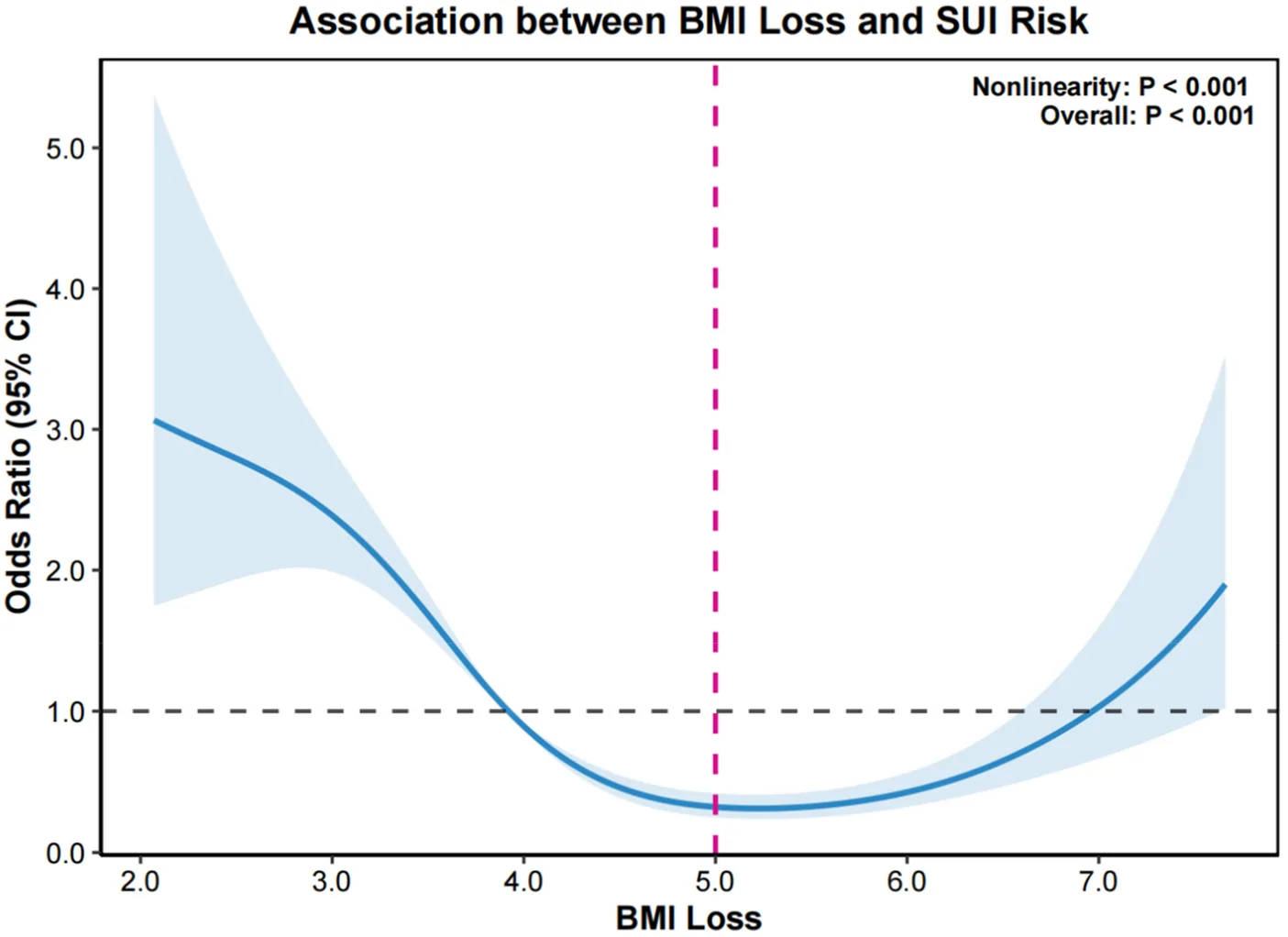
Association Between Postpartum BMI Loss and the Odds of Persistent SUI Modeled by RCS.

The RCS analysis demonstrated a significant nonlinear association between BMI loss and SUI risk (*P* for nonlinearity < 0.001), with the risk decreasing initially and subsequently increasing as BMI loss progressed, consistent with a U-shaped pattern (see Figure 6). To further characterize this potential nonlinearity between postpartum BMI loss and persistent SUI, a threshold effect analysis was performed using both linear and two-piecewise linear regression models (Table 3). Postpartum BMI loss was calculated as pre-pregnancy BMI minus BMI at 1 month postpartum, with higher values indicating greater BMI reduction by 1 month postpartum. Initially, a linear regression model indicated that greater BMI loss was significantly associated with a reduced likelihood of persistent SUI (OR = 0.643, 95% CI: 0.585–0.706, *P* < 0.001).

**Table 3:** Threshold effect analysis of BMI loss on SUI.

**Analysis Method**	**OR (95%CI)**	***P*-value**
Linear regression model	0.643 (0.585, 0.706)	<0.001
Two-piecewise linear regression model		
Below 5 kg/m^2^	0.362 (0.303, 0.429)	<0.001
Above 5 kg/m^2^	1.791 (1.39, 2.307)	<0.001
Threshold point	5 kg/m^2^	
Model comparison (LRT)		<0.001

BMI loss, body mass index loss; CI, confidence interval; OR: odds ratio; LRT, likelihood ratio test. Postpartum BMI loss was calculated as pre-pregnancy BMI minus BMI at 1 month postpartum. Threshold analysis was performed using a two-piecewise linear regression model. *P* < 0.05 was considered statistically significant.

However, a two-piecewise linear regression model revealed a significant threshold effect at 5 kg/m^2^ of BMI loss, as evidenced by a statistically significant likelihood ratio test comparing the two models (*P* < 0.001). Below this threshold, each 1 − kg/m^2^ increase in BMI loss was associated with a substantial decrease in the odds of persistent SUI (OR = 0.362, 95% CI: 0.303–0.429, *P* < 0.001). Conversely, above the 5 kg/m^2^ threshold, the direction of association reversed, with greater BMI loss being associated with higher odds of persistent SUI (OR = 1.791, 95% CI: 1.390–2.307, *P* < 0.001).

Among the 1,538 participants, 437 women had postpartum BMI loss greater than 5 kg/m^2^, accounting for 28.41% of the total cohort. This indicates that the subgroup above the identified threshold was not extremely small. Nevertheless, this threshold finding should be interpreted cautiously because postpartum BMI loss may be influenced by multiple unmeasured postpartum factors, including lactation, dietary intake, physical activity, postpartum recovery status, illness, and body composition changes.

## Discussion

This retrospective cohort study aimed to identify key risk factors associated with persistent postpartum SUI after basic PFMT and to develop a predictive model for early risk stratification and individualized intervention planning. Our findings highlight several independent risk factors for persistent SUI, including older maternal age, higher BMI, gestational diabetes mellitus, higher parity, perineal tears, cystocele, uterine prolapse, rectocele, and levator ani hiatus defects. Protective factors associated with SUI resolution included cesarean section delivery and a moderate level of postpartum BMI loss. We developed a clinically interpretable nomogram with good discriminatory performance (AUC = 0.813). A novel and particularly important finding was the complex, non-linear relationship between postpartum BMI loss and SUI risk, specifically a U-shaped association, suggesting that moderate BMI loss may be beneficial, whereas excessive BMI loss (>5 kg/m^2^) may be associated with an increased risk of persistent SUI.

The association between older maternal age and persistent SUI aligns with existing literature (27, 28). Aging leads to degenerative changes in pelvic floor muscles and connective tissues, including reduced collagen content, elasticity, and muscle strength, which may compromise pelvic floor structural support and reduce responsiveness to PFMT. Cumulative parity and multiple vaginal deliveries, often correlated with age, further exacerbate these vulnerabilities (29). This incremental risk underscores the need for early intervention and preventive strategies, particularly for older postpartum women (30).

Higher BMI emerged as a significant risk factor for persistent SUI, consistent with prior studies (27, 31, 32). Excess adiposity may increase intra-abdominal pressure and chronically overload the pelvic floor, leading to stretching and weakening of pelvic support structures (31). Obesity-related inflammation and metabolic dysfunction may further impair connective tissue integrity and pelvic floor function (33). Notably, we identified a non-linear association between postpartum BMI loss and persistent SUI. Restricted cubic spline and threshold effect analyses demonstrated a U-shaped relationship, with the lowest risk observed at approximately 5 kg/m^2^ BMI loss. When BMI loss was <5 kg/m^2^, greater BMI reduction was associated with a lower risk of persistent SUI, possibly because of reduced intra-abdominal pressure and mechanical load on the pelvic floor (34). However, when BMI loss exceeded 5 kg/m^2^, the risk increased again. This finding should be interpreted cautiously. Excessive postpartum BMI loss may reflect heterogeneous clinical and behavioral conditions, including breastfeeding-related weight change, nutritional status, physical activity, baseline adiposity, postpartum illness, socioeconomic factors, or loss of lean muscle mass, which were not fully captured in this retrospective dataset. One possible explanation is that excessive postpartum BMI loss may be associated with loss of lean muscle mass, inadequate nutritional support for connective tissue repair, and impaired recovery of pelvic floor strength and function. In addition, rapid changes in body composition may exceed the adaptive capacity of pelvic floor tissues during the postpartum recovery period (34, 35). These findings suggest that postpartum weight management should be balanced and individualized, and that rapid or excessive weight loss may not be beneficial for pelvic floor recovery. Nevertheless, this interpretation remains speculative and warrants confirmation in future studies with detailed assessment of lactation status, body composition, dietary intake, physical activity, and pelvic floor muscle function.

Gestational diabetes was identified as an independent risk factor for persistent SUI, corroborating previous research (36, 37). Potential mechanisms include microvascular complications, neuropathy affecting pelvic floor innervation, and increased risk of macrosomia, leading to greater birth trauma (38). Hyperglycemia may also impair collagen synthesis, weakening pelvic floor support structures. In addition, impaired glucose metabolism may affect tissue repair and muscle recovery during the postpartum period. These findings emphasize the importance of effective gestational diabetes management and postpartum monitoring to reduce long-term SUI risk (39).

Perineal tears have been reported as a potential risk factor for persistent SUI (37, 40). This association may be related to trauma to pelvic floor structures and possible involvement of the pudendal nerve, which may impair pelvic floor muscle function and reduce responsiveness to PFMT (40, 41). Notably, even less severe tears may be associated with subsequent pelvic floor dysfunction, underscoring the importance of appropriate perineal management and repair during delivery to minimize long-term sequelae (42). Conversely, cesarean section delivery was a strong protective factor (43) as it avoids direct trauma to pelvic floor structures during vaginal delivery (44). However, this association should not be interpreted as evidence that cesarean section causally prevents postpartum SUI. While pregnancy-related hormonal changes and increased intra-abdominal pressure can still contribute to SUI post-cesarean, the absence of delivery-related injury may partly explain the higher likelihood of symptom resolution (45). Given the observational design of this study, potential selection factors related to delivery mode should also be considered.

Furthermore, our study underscored the critical role of pelvic floor structural integrity, as evidenced by ultrasound findings. Baseline pelvic floor abnormalities such as cystocele, uterine prolapse, rectocele, and levator ani hiatus defects were identified as significant independent risk factors for persistent SUI (46). These findings are consistent with the understanding that compromised pelvic floor support, whether from direct injury or inherent weakness, reduces the effectiveness of basic PFMT by limiting the mechanical advantage of muscle contractions. The objective assessment via transperineal ultrasound (TPUS) provided anatomical information that may improve early risk stratification and help guide individualized rehabilitation planning, reinforcing the prognostic value of pelvic floor structural assessment for SUI resolution.

The nomogram developed in this study integrates these risk and protective factors, offering a practical tool for clinicians to assess SUI risk at the one-month postpartum visit. With an AUC of 0.813, the model demonstrates good discriminatory ability, enabling early identification of women at higher risk of persistent SUI after basic PFMT. In clinical practice, this model may support individualized rehabilitation strategies by identifying women who require closer follow-up, earlier pelvic floor rehabilitation, or further specialist assessment. For women with poor symptom improvement after basic PFMT, additional evaluation of contraction technique, adherence, and pelvic floor structural abnormalities may be warranted, followed by escalation to more intensive conservative treatment or referral to a specialist when appropriate. Such early risk stratification facilitates timely and targeted referral for advanced interventions, such as intensive supervised PFMT, biofeedback, electrical stimulation, or specialist consultation with a urogynecologist. This proactive approach may help reduce the psychosocial burden of chronic SUI, improve treatment adherence, and optimize healthcare resource allocation, particularly in resource-limited settings. Nevertheless, because the nomogram was developed and internally evaluated in a single-center retrospective cohort, external validation is required before it can be recommended for broad clinical use.

Importantly, this study was conducted within the framework of routine clinical practice, in which all participants received basic PFMT-based postpartum care. Specifically, each patient underwent an initial physician-guided instruction session, followed by weekly WeChat reminders and monthly outpatient reassessments. This management pathway reflects a pragmatic, real-world model of early conservative intervention. However, as the intensity, execution quality, and adherence to home-based PFMT could neither be fully standardized nor quantitatively captured in this retrospective study, some heterogeneity in actual training exposure likely remained. This variability should be taken into account when interpreting both the treatment outcomes and the generalizability of the predictive model. In particular, the model may be most applicable to clinical settings that employ similar postpartum PFMT management pathways. Further prospective multicenter studies with external validation are warranted to evaluate the model's performance in populations receiving different or more rigorously standardized rehabilitation protocols.

Although this study was motivated in part by the clinical burden of postpartum SUI, validated quality-of-life instruments were not routinely collected in the present dataset. Therefore, this study could not directly evaluate changes in quality of life. Any implications regarding quality-of-life improvement should be interpreted as clinically plausible rather than directly measured outcomes. Future studies should incorporate validated symptom severity and quality-of-life scales to more comprehensively evaluate patient-centered benefits.

### Strengths and limitations

This study's strengths include its large sample size, enhancing statistical power, and its focus on SUI resolution following basic PFMT, addressing a critical research gap. The use of TPUS for objective pelvic floor assessment adds rigor to our findings. The identification of a U-shaped relationship between BMI loss and SUI risk provides novel and clinically significant insights for counseling on postpartum weight management. The nomogram's development offers a practical, evidence-based tool for personalized SUI management.

Limitations include the retrospective design, which limits causal inference and is susceptible to unmeasured confounding. Data relied on medical record accuracy, and the single-center setting may reduce generalizability. External validation in diverse populations is needed to confirm the nomogram's applicability. Although all patients were managed within the same basic PFMT pathway, the actual intensity, execution quality, and adherence to home-based training could not be fully standardized or quantitatively captured in this retrospective study, which may have introduced heterogeneity into the observed outcomes. This treatment-exposure heterogeneity may have influenced the observed association between patient characteristics and persistent SUI. The study did not assess SUI severity, subtypes, or long-term outcomes beyond six months, leaving questions about recurrence or further improvement. In addition, the outcome was based on clinical symptom persistence during follow-up rather than standardized pad testing, validated severity questionnaires, or objective leakage quantification, which may have introduced outcome misclassification and limited assessment of disease severity. Factors such as psychological stress, diet, or physical activity were not fully captured. Other potentially relevant factors, including breastfeeding status, constipation, chronic cough, smoking, socioeconomic status, pelvic floor muscle strength, nutritional status, baseline adiposity, prior obstetric trauma severity, and detailed body composition, were also unavailable or incompletely recorded. Residual confounding is therefore likely. The unexpected association between excessive BMI loss and increased SUI risk requires further prospective studies to confirm and elucidate the underlying biological and physiological mechanisms. Finally, the model was internally validated but has not yet undergone external validation, and its clinical utility should therefore be considered preliminary.

## Conclusion

This study identified older age, higher BMI, gestational diabetes mellitus, higher parity, perineal tears, pelvic organ prolapse-related findings, and levator ani hiatus defects as risk factors for persistent postpartum SUI after basic PFMT, whereas cesarean delivery and moderate postpartum BMI loss were associated with better symptom resolution. The nomogram showed good predictive performance and may help identify women who require closer follow-up or intensified rehabilitation. The observed U-shaped association between BMI loss and persistent SUI suggests that postpartum weight management should be balanced and individualized. However, given the retrospective single-center design and lack of external validation, these findings should be confirmed in prospective multicenter studies.

## Data Availability

The data analyzed in this study is subject to the following licenses/restrictions: The datasets used and/or analyzed during the current study are available from the corresponding author on reasonable request. Requests to access these datasets should be directed to Jianxin Liu, liujianxin@zxhospital.com.
