# Risk factor analysis and predictive model for the alleviation of postpartum stress urinary incontinence: a retrospective cohort study

**DOI:** 10.3389/fgwh.2026.1965840

**Published:** 2026-09-09

**Authors:** Lingdan Guo, Jueying Li, Jia Lu, Sijiao Chen, Mengying Li, Jingzhou Yang, Li Xiao, Jian Shen, Jianxin Liu

**Affiliations:** 1 Department of Ultrasound, The Central Hospital of Wuhan, Tongji Medical College, Huazhong University of Science and Technology, Wuhan, China; 2 Department of Obstetrics and Gynecology, The Central Hospital of Wuhan, Tongji Medical College, Huazhong University of Science and Technology, Wuhan, China

**Keywords:** body mass index, nomogram, pelvic floor muscle training, postpartum stress urinary incontinence, quality of life, risk factors

Incorrect funding

The funder [Research and Development of a Multimodal Large Model for Multi-Organ Ultrasound Diagnosis], [insert Grant Number] to [26YJ11] was erroneously omitted.

The original version of this article has been updated.

In the published article, the Funding statement was incomplete.

The original Funding statement was:

“The author(s) declared that financial support was received for this work and/or its publication. Chinese Association of Plastic and Aesthetics, Grant Number: 202202036.”

The corrected Funding statement is:

“The author(s) declared that financial support was received for this work and/or its publication. Chinese Association of Plastic and Aesthetics, Grant Number: 202202036; Scientific Research Project “Research and Development of a Multimodal Large Model for Multi-Organ Ultrasound Diagnosis”, Grant Number: 26YJ11 (to Jianxin Liu).”

